# Therapies Targeting Immune Cells in Tumor Microenvironment for Non-Small Cell Lung Cancer

**DOI:** 10.3390/pharmaceutics15071788

**Published:** 2023-06-21

**Authors:** Wei Ye, Meiye Li, Kewang Luo

**Affiliations:** 1 School of Pharmaceutical Sciences, Southern Medical University, Guangzhou 510091, China; yeyewei2023@gmail.com (W.Y.); MeiyeLi2023@gmail.com (M.L.); 2 People’s Hospital of Longhua, Affiliated Longhua People’s Hospital, Southern Medical University, Shenzhen 518109, China

**Keywords:** NSCLC, TME, immune cells, immunotherapies

## Abstract

The tumor microenvironment (TME) plays critical roles in immune modulation and tumor malignancies in the process of cancer development. Immune cells constitute a significant component of the TME and influence the migration and metastasis of tumor cells. Recently, a number of therapeutic approaches targeting immune cells have proven promising and have already been used to treat different types of cancer. In particular, PD-1 and PD-L1 inhibitors have been used in the first-line setting in non-small cell lung cancer (NSCLC) with PD-L1 expression ≥1%, as approved by the FDA. In this review, we provide an introduction to the immune cells in the TME and their efficacies, and then we discuss current immunotherapies in NSCLC and scientific research progress in this field.

## 1. Introduction

Cancer is one of the leading causes of mortality worldwide [1], contributing to a reduction in life expectancy in many nations [2]. The Global Burden of Cancer 2020 (GLOBOCAN 2020) database from the World Health Organization estimates the most recent incidence and mortality rates for 36 varieties of cancer as well as cancer trends in 185 nations. This latest cancer report demonstrated that there were almost 19.3 million new cases of cancer and close to 10 million cancer-related deaths in 2020, and that by 2040 there would be 28.4 million new cancer cases worldwide [3,4]. The most recent edition of the International Agency for Research on Cancer (IARC) World Cancer Report shows that cancer is the primary cause of death among people aged 30 to 69 [5]. Lung cancer is the leading cause of cancer-related deaths and ranks second in terms of cancer incidence worldwide, with an estimated 1.8 million deaths (18%) in 2020 [4]. Non-small cell lung cancer (NSCLC) accounts for 85% of all lung cancer cases and is one of the most fatal tumors worldwide [6]. According to the Chinese National Cancer Center’s statistics for 2016, the number of lung cancer cases was about 787,000 and the number of deaths was about 630,000, making it the primary cause of mortality (21.7%) in China [7]. In recent years, the survival rate of NSCLC patients has increased due to the development of surgery, radiotherapy, and other treatments. However, the 5-year survival rate remains low, with a rate of 15% [8].

The tumor microenvironment (TME) is widely considered to be vital in dynamically modulating tumor growth and affecting therapeutic effectiveness, and numerous therapeutic approaches targeting components of the TME have been designed recently. The TME is defined as the cellular environment. It is composed of various cell types, such as cancer cells, immune cells, stromal cells, and cytokines as well as chemokines [9,10]. Much scientific research indicates that regulating a particular TME component can increase cancer survival rates. Garon et al., revealed that the combination of docetaxel and ramucirumab, which targeted vascular endothelial growth factor receptor-2 (VEGFR-2), could effectively prolong the lifetime of NSCLC patients [11]. Gocheva et al., illustrated that Tenascin-C, one of the components of the extracellular matrix, was much more prevalent in primary and metastatic lung cancers than in normal tissue, which was closely related to lung cancer patient survival [12]. Moreover, a study by Lambrechts analyzed the TME cell profile of lung cancer and discovered that a subpopulation of Tregs from NSCLC was related to a poor prognosis for lung adenocarcinoma [13].

Immune cells have been shown to be crucial in the TME. Tumor cells can develop a variety of immunosuppressive mechanisms, causing cancer cells to evade immune surveillance and undergo immune escape, resulting in tumor proliferation after treatment [14]. Different types of immune cells within the TME are able to activate an immune response through a number of interactions, and lead to a substantial impact on tumor progression and metastasis [15]. In the past few decades, a number of immunotherapies have already been used to treat different types of cancer. Immunotherapy is an essential part of cancer therapy, with better efficacy and fewer side effects than conventional chemotherapies. Rodriguez-Garcia demonstrated that CAR-T cell therapy, which was approved by the FDA for use in clinical treatment for B cell acute lymphoblastic leukemia, is an extremely successful therapy for hematological malignancies [16,17]. There is also increased promise for treatment of tumors with immunotherapies targeting Dendritic Cells (DCs), such as DC vaccines. DCs can induce tumor-directed immune responses in cancer patients by activating cytotoxic T cells [18]. The treatment of advanced NSCLC has undergone a fundamental change due to the advent of immunotherapy in recent years. The use of immune checkpoint inhibitors (ICIs) alone or in combination with chemotherapy has become the primary immunotherapy strategy for advanced or metastatic NSCLC without driver mutations [19]. The FDA has approved the use of ICI therapies such as nivolumab, pembrolizumab and atezolizumab as first-line therapy for NSCLC patients with high PD-L1 expression [20]. Nevertheless, a large proportion of patients still suffer from disease progression. 

In this review, we focus on the immune cells in the TME and tumor development. We also discuss the mainstream immunotherapies currently used to treat NSCLC and some novel therapeutic approaches used in the clinic, highlighting therapies that are under clinical trials or that have been approved.

## 2. Immune Cells in Tumor Microenvironment

**Figure 1:**
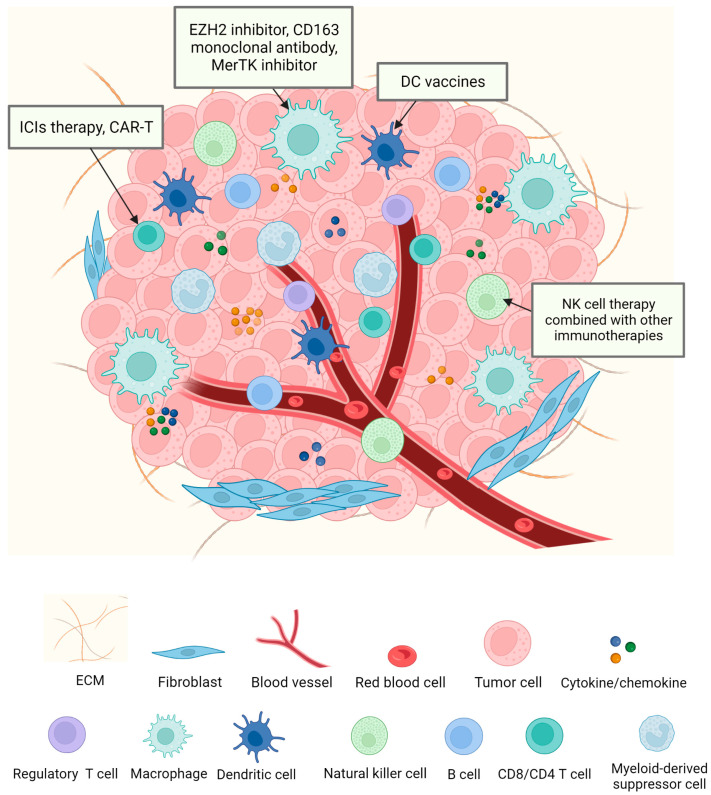
Tumor microenvironment and immunotherapies. The TME is composed of various cell types, such as cancer cells, immune cells, and stromal cells, and cytokines as well as chemokines. These growth factors and cytokines are essential for communication between the TME and other cell types. Due to the importance of immune cells in the TME and in tumor progression, a number of immunotherapies have been developed against tumors in recent years. Immunotherapies that have been approved for use or that are undergoing clinical trials focus on TAMs, DCs, T cells, and NK cells as their main targets.

Immune cells are a fundamental part of the TME and are essential for both pro- and antitumor immune responses [21]. The cells of the immune system include both innate immune cells, such as macrophages, neutrophils, mast cells, DCs, and MDSC and NK cells, and adaptive T and B lymphatic immune cells [22]. Early studies using immunohistochemistry to identify immune cells in NSCLC found that NSCLC tumors contain multiple types of immune cells, including T cells, B cells, macrophages, NK cells, and DCs, and many studies have demonstrated a crucial link between immune cells and the survival of NSCLC patients [23,24]. It is clear that immune cells are a non-negligible presence in the TME. Most importantly, the immune system is normally capable of monitoring, recognizing, and destroying cancerous cells [25]. However, due to their close proximity to tumor cells, or as a result of chemokine and cytokine communication, the immune cells within the TME are able to evade identification and annihilation by the host immune system [26]. The occurrence of lung cancer and the entire process from early lung cancer to metastatic lung cancer are considered to depend on the immune escape mechanism [27,28]. Therefore, the role that immune cells play in the TME and the mechanisms by which they influence tumor growth deserve to be discussed. In the following part, we introduce the main immune cells in the TME, including T cells, tumor-associated macrophages, dendritic cells, and natural killer cells (Figure 1).

### 2.1. T Cells

T cells are an essential part of the immune system, which is responsible for defending cells against cancer and viruses [29]. T cells can be further divided into three subpopulations: cytotoxic T lymphocytes (CTLs), T helper cells (Ths), and Tregs [30]. CTLs are the cells in the TME with direct tumor-killing power, Ths cells help to postpone tumor growth, and Treg cells are typically associated with poor outcomes in tumor patients.

CTLs (which include cytotoxic CD8^+^ T cells and CD4^+^ T cells) in the TME can directly destroy malignant tumors. As major killers of pathogens and cancers, CD8^+^ T cells are the preferred immune cells in the fight against cancer [31,32]. The presence of cytotoxic CD8^+^ T lymphocytes is also linked to better outcomes in NSCLC patients [33]. When CD8^+^ T cells develop into cytotoxic T cells, naive CD8^+^ T cells are connected to the peptide major histocompatibility complex (MHC) on antigen-presenting cells (APCs) via their T-cell receptors (TCRs), and are activated by co-stimulatory signals and extracellular cytokines. These CD8^+^ T cells subsequently become effectors, and their activation enables them to attack cancer cells directly [34]. In addition to actively destroying tumor cells, CD8^+^ T cells have the capacity to prevent tumor angiogenesis by secreting interferon-γ (IFN-γ) [35]. However, prolonged antigenic stimulation transforms CD8^+^ T cells in the TME into a T cell-depleted hypoactive state (including progressive loss of effector function and persistent expression of inhibitory receptors), which prevents the tumor cell-killing function of CD8^+^ T cells from proceeding normally [36,37]. Apart from CD8^+^ T cells, CD4^+^ T cells are essential for tumor elimination. CD4^+^ T cells can directly contribute to CD8^+^ T cell activation, support CD8^+^ T cells in the formation of memory CTL, and enhance CD8^+^ T cell proliferation by generating interleukin(IL)-2 [38,39]. Furthermore, CD4^+^ T cells can help activate CD8^+^ T cells by delivering tumor antigens to CD8^+^ T cells or by triggering the production of cytokines and suppressor molecules [40]. 

Ths cells are generated by cytokine-specific polarization or TCR-signaling activation in the TME, and they can assist CD4^+^ T lymphocytes in producing cytotoxicity and destroying tumor cells [41]. Th1 and Th9 cells in the TME boost CD8^+^ T cells’ ability to fight tumors, mainly by secreting IL-4 and IFN-γ, which are linked to a favorable prognosis in various cancer types [9,42]. 

Tregs normally inhibit autoimmunity by restricting the immune system’s reaction to autoantigens [43]. However, Tregs in the TME can promote cancer growth by reducing the function of lymphatic and bone marrow immune cells [44]. Many studies have reported that Treg cells in the TME suppress effector T cells directly by generating higher levels of cytokines than those associated with T-cell activation, resulting in immunosuppressive TME and the progression of tumors [45,46,47,48]. On the other hand, Treg cells are capable of promoting tumor growth and metastasis by secreting growth factors, interacting with stromal cells, and undergoing immune escape [43]. A higher Treg-to-effector T-cell ratio in NSCLC tumor tissue has been linked to a worse prognosis [49].

### 2.2. Tumor-Associated Macrophages (TAMs)

The majority of immune cells that infiltrate the TME are tumor-associated macrophages, which influence tumor angiogenesis and metastasis and are related to a poor prognosis for various cancers [50,51]. In the TME, TAMs may be differentiated into classical activated macrophages (M1) or, alternatively, activated macrophages (M2) [52,53]. Phenotype M1 is able to directly kill tumor cells by producing nitric oxide and reactive oxygen species, or by secreting pro-inflammatory cytokines such as tumor necrosis factor-α (TNF-α), IL-6, and IFN-γ [50,54,55]. Additionally, through the process of antibody-dependent cell-mediated cytotoxicity (ADCC), M1 has the capacity to eradicate malignant cells [56]. In contrast, M2 stimulates angiogenesis, tissue healing, and cancer-cell proliferation by secreting anti-inflammatory cytokines such as IL-4, IL-1, and VEGF [57,58,59]. As macrophages are highly plastic in the TME, they convert between M1 and M2 in response to microenvironmental factors (cytokines, growth factors, chemokines, inflammation, hypoxia, and infection), thereby inhibiting the antitumor response and producing immunosuppressive TME. This is a process known as macrophage polarization [52,59,60]. It has been found that tumor cells express significant amounts of colony-stimulating factor 1, C-C pattern ligand 2 (CCL2), and monocyte chemotactic protein-1, something which contributes to TAMs’ function in tumor development [61]. At the same time, TAMs activate tumor cells by activating transcription factors (STAT3 and NF-κB) to produce VEGF and matrix metalloproteinases (MMPs) to promote tumor angiogenesis [51,62]. As the relationship between TAMs and cancer becomes clearer, TAMs have become a desirable target for the development of novel immunotherapies. It has been demonstrated that activation of TAM receptors reduces resistance to ICI treatment in NSCLC patients, indicating that the combination of the two strategies may be beneficial for these patients [63]. 

### 2.3. Dendritic Cells (DCs)

As the most powerful APCs, DCs serve as central regulators of the adaptive immune response and connect innate and adaptive immunity [64,65,66]. DCs are inherently plastic and can be stratified into a variety of subtypes [15]. According to their phenotypes and their functions in the TME, DCs are typically divided into two major subgroups: plasmacytoid DCs (pDCs) and myeloid DCs (mDCs) [67,68]. 

A primary function of pDCs is to produce IFN-Ⅰ, which is needed to counteract the antiviral immune response [65,69]. It has also been suggested that pDCs may have potential as APCs due to the MHC-Ⅱ and co-stimulatory molecules they express at steady state [70]. In the TME, the function of pDCs is controversial. Tumor-infiltrating pDCs are reported to produce immunosuppressive and carcinogenic effects; however, due to their ability to secrete INF-Ⅰ and TNF-α, pDCs are potential therapeutic targets for combating cancer [71,72]. 

In the TME, conventional DCs (cDCs), as the typical representatives of mDCs, can be further subdivided into cDC1 and cDC2 spectrums [73]. cDC1 is regulated by transcription factors IRF8, ID2, and BATF3, while cDC2 is regulated by transcription factors IRF4, ID2, ZEB2, and Notch2 [74]. cDC1 is able to cross-present antigens and present exogenous antigens to CD8^+^ T cells on MHC-I, activating CD8^+^ T cells and making them the primary mediators of antitumor immunity [75]. Hence, cDC1 has the unique ability to induce intracellular infections and encourage the immune system to reject malignant cells [15,65,76]. The cDC2 exhibits enhanced presentation of the MHC II antigen. In contrast to cDC1, cDC2 presents only exogenous antigens without cross-presentation and activates CD4^+^ T cells selectively, making it the most effective activation and expansion agent for CD4^+^ T cells [77,78]. 

It has been shown that immunogenic DCs secrete substantial quantities of inflammatory cytokines, including TNF-α, IL-12, IL-6, and IL-8, which may improve the clinical outcomes of tumor patients [79,80]. Despite this, the uptake of antigen by DCs in the TME is often insufficient. The phenomenon is caused by aberrant expression of some tumor-derived chemokines on DCs within the TME, resulting in the suppression of DC invasion [67]. The maturation state of DCs and specific markers expressed by different subgroups of DCs have been considered significant indicators of prognosis and prediction in human cancer. DC-based vaccines have been developed to induce and maintain immune responses [78]. Consequently, the use of DC therapy in conjunction with other immunotherapies to reduce immunosuppressive TME can serve as a future treatment strategy for cancer. Zhong et al., found that chemotherapy combined with a DC vaccine significantly inhibited tumor growth in a mouse model of NSCLC [81].

### 2.4. Natural Killer Cells (NK Cells)

As innate lymphocytes, NK cells can stimulate the immune system to destroy cancer cells [82]. It has been demonstrated that NK cells can kill tumors by direct cytotoxic killing of target cells [83]. Besides, NK cells can inhibit tumor growth by inducing pro-inflammatory cytokines, which include IFN-α, TNF-α, and granulocyte macrophage colony-stimulating factor [84].

NK cells have been divided into two distinct subpopulations based on CD56 expression levels, CD56^bright^CD16^−^ and CD56^dim^CD16 [85]. When CD56^bright^ NK cells are exposed to pro-inflammatory cytokines including IL-12, IL-15, and IL-18, they produce additional cytokines to regulate adaptive immunity and cause cytotoxicity [86,87]. CD56^dim^ NK cells can directly mediate the death of infected and cancer cells via cytotoxicity and cytokine production [86,88]. NK cells may also eliminate MHC class I tumors and carry out antigen presentation on their own, creating a connection between innate and adaptive immunity [89].

NK cells are associated with both the killing of tumor cells and the prevention of cancer progression. However, NK cell toxicity is decreased in the TME [9,90]. A change in angiogenesis may be responsible for this, as well as insufficient access to nutrients and oxygen. Many studies have reported that hypoxia and the secretion of transforming growth factor-β (TGF-β) and other cytokines in the TME limit the activity of NK cells [91,92,93]. TGF-β can reduce the antitumor effect of NK cells by transforming peripheral CD4^+^ T cells activated into Treg cells [92,94]. One study found that delivering TGF-β inhibitors and selenocysteine to breast cancer cells can reduce TGF-β in the TME, improve NK cell activity, and increase antitumor capability, suggesting that NK-targeted immunotherapies may be promising [95]. 

### 2.5. Other Types of Immune Cells That Affect Tumor Development

Apart from the immune cells discussed above, neutrophils, MDSCs, and B cells also play crucial roles in the TME.

Neutrophils, the most abundant innate immune cells in the bone marrow and peripheral blood, serve a complex and important function in cancer development [96]. In many cancer patients, neutrophil peripheral blood counts are elevated, and neutrophil-to-lymphocyte ratios are independent prognostic factors [97,98]. In the TME, neutrophils produce MMP-9 to stimulate angiogenesis, boosting cancer growth and offering extra immune-escape pathways for cancer cells [97]. The neutrophil extracellular trap (NET) can promote tumor progression, metastasis, invasion, and angiogenesis [99,100]. Several studies have shown that mice with lung cancer, breast cancer, and chronic myeloid leukemia are more likely to release NET than healthy mice [100,101,102]. 

MDSCs are a heterogeneous cell population composed of progenitor cells derived from bone marrow, immature macrophages, immature granulocytes, and immature DCs [103]. As part of the TME, they promote tumor angiogenesis and metastasis [104]. In Hinshaw’s study, it was found that MDSC could be stimulated in the TME and released chemokines including NO, CCL3, and CCL4, which inhibited innate and adaptive immune responses [15]. In addition, MDSCs promote epithelial mesenchymal transition by secreting IL-6, which increases angiogenesis and speeds up tumor progression [105,106]. 

Tumor-infiltrating B cells are responsible for antibody production, antigen presentation, and cytokine secretion in tumors, as well as for complementing T-cell-mediated antitumor immunity [9,107]. Recent studies demonstrated that B cells in the TME have prognostic value in various tumors [108,109]. 

## 3. Target Immune Cells against NSCLC

Given the crucial roles of immune cells in immunomodulation and tumor progression in the TME, strategies to target immune cells in the TME hold significant promise. In the past few decades, a number of immunotherapies have already been used to treat different types of cancer. Current research indicates that immunotherapies targeting ICIs when utilized in NSCLC patients have exhibited promising results in terms of five-year survival and progression-free survival (PFS) when compared with previous treatments [110,111]. 

In the following part of our review, the immunotherapies (Figure 1) used in NSCLC in clinical trials and in scientific research are discussed.

### 3.1. Target T Cells against NSCLC

**Figure 2:**
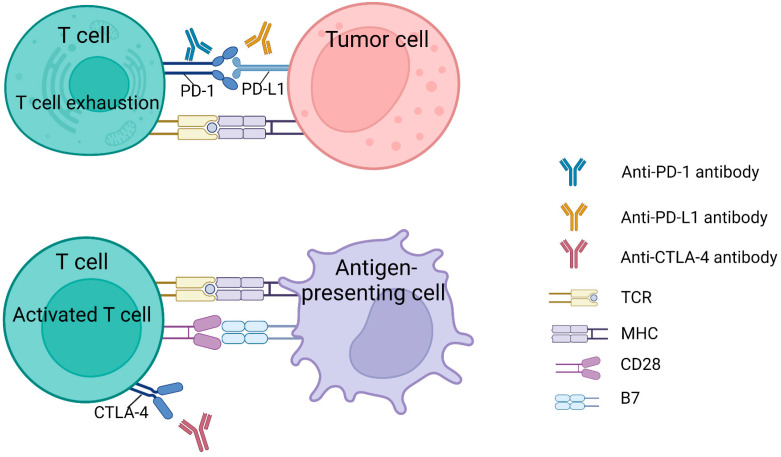
Two of the most common targeting pathways for ICIs (anti-CTLA-4 and anti-PD-1/PD-L1). PD-1 receptor on T cells leads to T cell exhaustion by binding to its ligand PD-L1 on tumor cells. Targeted antibodies that block PD-1 or PD-L1 prevent T cell exhaustion and maintain the ability of T cells to destroy tumors. T cells can be activated through two primary mechanisms. T cell receptors (TCRs) on antigen-presenting cells (APCs) present antigens with the major histocompatibility complex (MHC) and activate T lymphocytes. Additionally, the binding of CD28 on T cells and B7 on APCs can result in T-cell activation and antitumor activity. CTLA-4 antigen limits T cell-activation by competing for CD28 binding to B7 on the T cell surface, resulting in a decrease in T cell antitumor capacity. Therefore, the use of anti-CTLA-4 drugs can prevent the competitive behavior of CTLA-4 antibodies and support normal T cell-activation.

T cells are usually recognized as the most essential immune cells in attacking malignancies [112]. Among T-cell-targeted immunotherapies, treatments using ICIs have undergone the most extensive research and been approved by the FDA. This strategy keeps the antitumor immune response in the TME, bringing a new era of cancer immunotherapy [113,114]. An ICI is an immune checkpoint protein that maintains the ability of T cells to destroy tumors by binding to their partner proteins. Immune checkpoint molecules include CTLA-4, PD-1, etc. (Figure 2), and their role is to maintain an immune balance and prevent an immune response from attacking normal tissues and cells in the body. Immunotherapy drugs are mainly monoclonal antibodies, which can block immunosuppressive effect, allowing T cells to recognize and attack cancer cells, thereby enhancing immune responses, by binding with their partner proteins. These drugs (antibodies) are preferentially designed as ICIs [115]. The main agents undergoing clinical trial and research are nivolumab and pembrolizumab (PD-1 antibody), atezolizumab (PD-L1 antibody), and ipilimumab (CTLA-4 antibody) [116].

According to scientific studies, ICIs have a significant impact on patients’ prognoses, and have become the first-line therapy for most individuals with advanced lung cancer [117,118,119]. PD-1 receptor is mainly expressed on activated T cells, and by adhering to ligands PD-L1 and PD-L2, it suppresses the antitumor activity of T cells [120,121]. Expression of PD-L1 is widespread in cancer cells and elevated in the TME, leading to suppression of the antitumor immune response by limiting the signals that activate T cells [112,121]. Nivolumab is an antibody against PD-1. As demonstrated in a clinical trial (NCT01642004), among patients with metastatic NSCLC (mNSCLC) (stage III patients) for whom platinum-based chemotherapy had failed, improved overall survival (OS) and substantially improved PFS were seen in individuals treated with nivolumab [122]. An additional study comparing the efficacy of nivolumab and docetaxel (a chemotherapy drug) in patients with mNSCLC (NCT01673867) also discovered positive results for nivolumab [123]. As a result, nivolumab is the first ICI targeting PD-1 to receive FDA approval for use in the treatment of patients with mNSCLC. Pembrolizumab is also an anti-PD-1 antibody. Patients with mNSCLC who participated in a randomized, phase III trial (NCT02142738) demonstrated significant improvements in 6-month PFS, OS, and objective response rate (ORR) when compared with platinum-based chemotherapy [124]. Furthermore, a phase III trial (NCT03850444) comparing pembrolizumab to platinum-based chemotherapy in patients with advanced or mNSCLC found that pembrolizumab had a meaningful effect in individuals whose PD-L1 expression was higher than 50% [125]. The FDA has approved pembrolizumab as a first-line treatment for patients with PD-L1 levels ≥ 1%, and these results have led to the authorization of pembrolizumab by the European Medicines Agency (EMA) as a first-line treatment for patients with PD-L1 levels ≥ 50% [20]. Atezolizumab, a PD-L1 antibody, has also been approved by the FDA and the EMA as a single-agent treatment for patients with mNSCLC with high PD-L1 expression [20,126], having shown significant benefits in treating patients with NSCLC in clinical trial NCT02409342 (Table 1).

Moreover, ICIs includes antibodies against CTLA-4, which inhibits T-cell proliferation by competing binding with CD28 on the cell surface [127]. Since the antitumor effect of CTLA-4 antibodies is mainly related to T-cell initiation in lymph nodes and the TME, this may offset some ICI resistance [120,128,129]. Ipilimumab is a therapeutic antibody targeting CTLA-4 [130]. A trial (NCT02477826) has been carried out on patients with stage IV NSCLC to compare the efficacies of three different strategies: nivolumab alone, the combination of nivolumab and ipilimumab, and the combination of chemotherapy with nivolumab plus platinum [131]. Recent findings from this trial indicated that the combination of nivolumab and ipilimumab enhanced OS for those patients with PD-L1 expression levels of more than 1%, suggesting that the ICI two-drug combination could be considered as a first-line therapy for NSCLC. Meanwhile, the results of this trial also implied a positive outcome from the combination of chemotherapy and anti-PD-1 drugs [132]. In addition, in a clinical trial of patients with metastatic squamous NSCLC treated with carboplatin-paclitaxel/nab-paclitaxel with or without pembrolizumab (NCT03875092), it was found that PFS and OS improved significantly when anti-PD-1 medications were taken in conjunction with chemotherapy [133] (Table 1). Combination therapy with ICIs has been shown to have great potential in terms of antitumor response. Apart from chemotherapy and ICIs, many studies indicate that inhibitors targeting IDO, VEGF, lymphocyte activation3 and T-cell immunoglobulin mucin 3 may also be considered as future combination options [134,135,136,137]. 

In addition to the immunotherapy of ICIs against NSCLC, chimeric antigen receptor-modified T cells (CAR-T cells, a type of genetically engineered T cells) treatment is another very rapidly developing form of cellular immunotherapy [138]. Synthetic CAR vectors are genetically engineered to recognize and bind specifically to tumor cell surface antigens (such as CD19) to achieve antitumor effects in this therapy [139,140]. However, the presence of tumor-associated antigens (TAAs) in NSCLC has been heterogeneous in terms of both intensity and distribution, severely limiting the clinical efficacy of CAR-T therapy [141]. 

Recently, understanding of the molecular typing of NSCLC has been significantly enhanced, because of research into the causative genes of NSCLC patients [142,143]. It is clear that the molecular typing of NSCLC can be classified into three groups. The first category refers to genes with clinical relevance, such as KRAS, HER2, ROS1, TP53, BRAF, and NTRK, and relevant medications targeting these genes are currently being researched [142,144]. Several studies have demonstrated that patients with mutant NSCLC, such as KRAS, BRAF, and TP53, can benefit from ICIs [145,146,147]. The second category consists of treatable mutation-driver genes. The current focus of therapies based on mutation-driver genes in NSCLC are epidermal growth factor receptor (EGFR) and anaplastic lymphoma kinase (ALK) [142]. Because of the mutations in EGRF or ALK, the efficacy of ICI treatment in these NSCLC patients is not obvious [19,148]. Many targeted medicines that target the driver genes of EGFR and ALK have had some success [143]. The third category includes biomarkers that have some association efficacy, such as PD-L1 and TMB [149,150]. In NSCLC patients with mutations in EGFR, the PD-L1 expression is predominantly reduced. Consequently, anti-PD-1 ICIs are ineffective in this group of patients [151]. However, EGFR-tyrosine kinase inhibitors (EGFR-TKIs) have been developed to treat NSCLC patients. First-generation EGFR-TKIs, such as gefitinib, erlotinib, and ecotinib, have been shown to be effective in treating patients with EGFR-sensitive mutant NSCLC. Afatinib is a second-generation EGFR-TKI that was approved by the FDA and the EMA in 2013 used to treat adults with advanced, EGFR mutation-positive NSCLC [152]. However, long-term use of EGFR-TKI would result in drug resistance and the loss of the original therapeutic effect [142]. Osimertinib, a third-generation EGFR-TKI, was developed to treat NSCLC patients who acquired the EGFR T790M resistance mutation [153]. Due to the drug resistance of EGFR-TKIs, future strategies must be developed to cope with resistance to next generation EGFR-TKIs, including conventional combinations of EGFR-TKIs and other agents and treatments combined with ICIs.

Although ICIs targeting PD-1 and CTLA4 have been shown to be beneficial in mNSCLC, the efficiency in NSCLC is, however, only 8–30% [154]. Emerging evidence indicates that driver oncogenes have different effects on the TME that influence the potential benefit from treatment with ICIs. For instance, patients harboring BRAF, KRAS or TP53 co-mutations benefit from ICIs [155,156]. However, certain patient populations do not respond to these drugs targeting ICIs [112]. Further strategies to enlarge the efficacy of ICIs are to synergistically combine them with CAR-T, EGFR-TKIs and other agents.

**Table 1:** Relevant immunotherapies for the treatment of NSCLC.

ClinicalTrials.gov Identifier	Target	Drug	Study Design	Cancer Types	Phase	Status	Enrollment	Outcomes	References
NCT01642004	T cells, PD-1	Nivolumab	Nivolumab vs. Docetaxel	Advanced or metastatic squamous cell NSCLC	Ⅲ	Completed	352	OS↑	[122]
NCT01673867	T cells, PD-1	Nivolumab	Nivolumab vs. Docetaxel	Metastatic non-squamous NSCLC	Ⅲ	Completed	792	OS↑	[123]
NCT02142738	T cells, PD-1	Pembrolizumab	Pembrolizumab vs. Paclitaxel + Carboplatin vs. Pemetrexed + Carboplatin vs. Pemetrexed + Cisplatin vs. Gemcitabine + Carboplatin vs. Gemcitabine + Cisplatin	Metastatic NSCLC	Ⅲ	Completed	305	PFS↑	[124]
NCT03850444	T cells, PD-1	Pembrolizumab	Pembrolizumab vs. Carboplatin + Paclitaxel + Pemetrexed	PD-L1 positive advanced or metastatic NSCLC	Ⅲ	Active, not recruiting	262	Undergoing	[125]
NCT02409342	Tumor cells, PD-L1	Atezolizumab	(Carboplatin/Cisplatin) + (Pemetrexed/Gemcitabine) vs. Atezolizumab	Stage IV non-squamous or squamous NSCLC	Ⅲ	Completed	572	OS↑, PFS↑	[126]
NCT02477826	T cells, CTLA-4	Ipilimumab	Nivolumab vs. Nivolumab + Ipilimumab	Stage IV or recurrent NSCLC	Ⅲ	Active, not recruiting	2748	Undergoing	[131]
NCT03875092	T cells, PD-1	Pembrolizumab	Pembrolizumab + Chemotherapy vs. Chemotherapy	Metastatic squamous NSCLC	Ⅲ	Active, not recruiting	125	Undergoing	[133]
NCT05467748	TAMs, EZH2	Tazemetostat	Tazemetostat + Pembrolizumab	Advanced NSCLC	Ib/II	Not yet recruiting	66	Undergoing	[157]
NCT05094804	TAMs, CD163	OR2805	OR2805 vs. OR2805 + PD-1 inhibitor	Advanced malignancies	I/II	Recruiting	130	Undergoing	[158]
NCT04762199	TAMs, MerTK	MRX-2843	Osimertinib + MRX-2843	Advanced EGFR mutant NSCLC	Ⅰ	Recruiting	69	Undergoing	[159]
NCT00103116	DC	Autologous dendritic cell cancer vaccine	Autologous dendritic cell cancer vaccine	Stage I, Stage II, or Stage III NSCLC	II	Completed	32	Safety, no related adverse events	[160]
NCT02808416	DC	Personalized cellular vaccine	Personalized cellular vaccine	Patients with brain metastases from solid tumors	Ⅰ	Completed	10	Induced specific CD4^+^ and CD8^+^ T cell responses	[161]
NCT02843204	NK cells	Pembrolizumab and NK immunotherapy	Pembrolizumab + NK immunotherapy vs. Pembrolizumab	Malignant solid tumor	I/II	Completed	110	OS↑, PFS↑	[162]
NCT02843815	NK cells	NK immunotherapy and cryosurgery	Cryosurgery + NK immunotherapy vs. Cryosurgery	Advanced NSCLC	I/II	Completed	30	Relief degree↑	[163]
NCT02118415	NK cells	Hsp70-peptide TKD/IL-2 activated, autologous NK cells	(Hsp70-peptide TKD/IL-2 activated, autologous NK cells) vs. Control group	NSCLC Stage IIIA/B	Ⅱ	Suspended	90	Undergoing	[164]

↑ means an improvement in the outcomes.

### 3.2. Target TAMs against NSCLC

Crosstalk between TAMs and other cells in the TME results in an immunosuppression that promotes tumor cell proliferation, angiogenesis, tumor metastasis, and the development of cancer-therapy resistance [165]. It is widely known that macrophage polarization may cause a direct switch between M1 and M2 in the TME, while subtype M1 could kill tumor cells and M2 can promote tumor progression [52]. Small molecule inhibitors or monoclonal antibodies against TAM signaling can consequently inhibit the progression and metastasis of cancer [129]. Recently, the main immunotherapies targeting TAMs and undergoing clinical or scientific trials are tazemetostat, OR2805, and MRX-2843.

Tazemetostat, an enhancer of zeste homolog 2 (EZH2) inhibitor, has been used individually or in combination with ICIs against advanced NSCLC in clinical trials. It has been demonstrated that EZH2 could stimulate the expression of CCL5 and result in the recruitment of macrophages and the invasion of lung cancer [166]. When EZH2 is inhibited, the quantity of M2 decreases, thereby preventing the progression of lung cancer [166]. An open-label, single-arm, phase Ib/II clinical trial (NCT05467748) is underway, in which pembrolizumab in combination with tazemetostat is used to treat patients with advanced NSCLC who have relapsed following initial or second-line therapy (Table 1) [157].

OR2805 is a monoclonal antibody against CD163. CD163 is one of the biomarkers of M2 macrophage polarization, which can inhibit PD-1 and PD-L1 signaling and stimulate macrophage polarization to the M1 phenotype, resulting in enhanced antitumor activity [167,168]. Therefore, OR2805 can be used as a monotherapy or in combination with PD-1 inhibitors to treat patients with advanced solid tumors, and is a valuable targeted TAMs therapy. An open-label, multicenter, first-in-human dose escalation and extension phase I, II trial (NCT05094804) to verify the safety, tolerability, pharmacokinetics, pharmacodynamics, and preliminary anticancer activity of OR2805 is at the recruitment stage, with individuals with NSCLC being expected to enroll (Table 1) [158]. 

MRX-2843 is a mer tyrosine kinase (MerTK) inhibitor. MerTK is a macrophage-specific tyrosine kinase receptor (RTK) that can induce tumor immunological tolerance by inducing macrophage death, polarizing macrophages towards the M2 phenotype, and inhibiting the secretion of inflammatory factors [169,170]. In contrast, blocking MerTK signaling repolarizes macrophages to M1 phenotypes and enhances antitumor immunity [171]. MerTK antibodies stimulate T-cell activation and are synergistic with anti-PD-1 and anti-PD-L1 treatments [172]. Blocking the macrophage MerTK protein may increase antitumor immunity and induce tumor immunogenicity, a promising method for treating cancer. An ongoing phase Ⅰb safety and pharmacodynamic study is being conducted with MRX-2843 in conjunction with osimertinib in patients with advanced EGFR-mutated NSCLC (NCT04762199) (Table 1) [159]. Melittin (MEL-dKLA) is a promising option for NSCLC patients because it selectively induces cell death in M2 macrophages in vitro without interacting with the normal function of other cell types [173,174]. In addition, a preclinical study showed that the construction of targeted nano-micelles (called ‘nano-dandelion’) for simultaneous delivery of curcumin (Cur) and baicalin (Bai) was effective in overcoming tumor resistance, which resulted in the transforming of TAMs to tumor-killing M1-type macrophages, indicating an adjuvant therapy for tumors [175]. 

Due to the high level of macrophage infiltration, the prognosis for NSCLC patients is poor. Macrophage polarization promotes the cancer development in the TME, thus targeting TAMs has emerged as a promising therapeutic strategy and has led to the development of numerous relevant drugs undergoing clinical trials.

### 3.3. Target DCs against NSCLC

As the primary APCs, DCs trigger antigen-specific CD8^+^ T lymphocyte activation, which initiates innate and adaptive immune responses that are capable of killing tumors [176,177]. Usually, DC-targeted treatments include autologous DC vaccination and individualized cellular vaccination. In the majority of studies, patients are vaccinated with DCs via numerous subcutaneous injections of primed DCs [177]. Moreover, combination therapy with DC and cytokine-induced killer cells (DC-CIK) has been confirmed to be an effective treatment for NSCLC patients.

In a clinical trial (NCT00103116), an autologous DC vaccine was involved in the treatment of individuals with stage I, II, and III NSCLC. Specific T-cell activity in the blood was compared before and six months after vaccination. As a result of the trial, there are 22 out of 32 patients who demonstrated an immune response to the vaccine six months after vaccination, and 20 patients out of 32 who were still alive five years after dual vaccination. These pieces of evidence suggest that the DC vaccine has a favorable safety profile [160]. Moreover, one study was conducted in ten patients with tumor brain metastases (BM) (NCT02808416), five of whom were NSCLC patients. The trial was to examine the security and effectiveness of an individualized cellular tumor vaccination (DC vaccine) in patients with BM [161]. These patients had a good OS rate and an excellent objective response; however, due to the limited number of samples, additional clinical trials are required in the future [178]. Details of the above two tests are shown in Table 1. Based on the outcomes of the aforementioned trials, DC vaccines have demonstrated significant potential for treating NSCLC patients. 

In mouse models, DC vaccination was found to be effective when used in combination with chemotherapy and radiotherapy for the treatment of NSCLC [81,179]. Studies have compared the combined effects of chemotherapy or radiotherapy, DC vaccination, and cytokine-induced killer cells (CIK) in humans [180,181,182,183]. Consequently, a combination therapy consisting of DC-CIK may have a stronger antitumor effect. Clinical trials have shown that DC-CIK immunotherapy is effective in controlling the disease, improving immune function, and delaying the progression of advanced NSCLC without increasing adverse effects, indicating a promising future in the treatment of NSCLC patients [184].

DC-related immunotherapy for NSCLC patients focuses on DC vaccines and DC-CIK therapy. The safety of these treatments has been recognized, but their exact efficacy still needs to be further determined.

### 3.4. Target NK Cells against NSCLC

NK cells can destroy tumor cells directly or indirectly through the release of pro-inflammatory factors [83,185]. Many researchers have discovered that CD56^bright^ CD16^−^ NK cells that release high levels of cytokines (such as VEGF, placental growth factor, and IL-8/CXCL8) which promote tumor neoangiogenesis exhibit low cytotoxicity in the lung TME [89,185,186]. In addition, recent research has demonstrated that NK cells can identify and eliminate cancer progenitor cells in solid tumors, and have a beneficial effect on lung cancer [187,188]. 

Immunotherapies targeting NK cells are mostly utilized as an additional therapy with other treatments and are seldom used alone in clinical studies. It has been revealed that the mixture of NK and T lymphocytes cells (NKTm) improves OS and 2-year survival among patients with NSCLC [189]. In addition, NK immunotherapy in combination with cryosurgery or the PD-1 representative drug Pembrolizumab is currently under research. Hsp70-peptide TKD/IL-2 activated autologous NK cells are being considered as a new treatment for NSCLC patients after demonstrating their potential to treat cancer in preclinical models.

In a clinical trial (NCT02843204), NK immunotherapy combined with ICI pembrolizumab was used to treat patients with advanced relapsed NSCLC. Results from the study indicated that pembrolizumab combined with NK cell therapy improved OS and PFS in patients with previously treated advanced PD-L1 NSCLC without causing any serious adverse effects compared to pembrolizumab alone, showing significant potential for NK cell immunotherapy (Table 1) [162,190]. In a separate trial (NCT02843815), the safety and efficacy of cryosurgery combined with allogeneic NK cell immunotherapy for advanced NSCLC were initially determined (Table 1) [163,191]. Preclinical models demonstrated that the combination of NK cells and anti-PD-1 therapy provides long-term tumor control with a substantial infiltration of CD8^+^ T cells and NK cells [192]. A combination-therapy study using anti-PD-1 antibodies and autologous NK cells has made significant progress in view of the enormous potential NK cells have demonstrated in tumor therapy.

Currently, a trial (NCT02118415) is taking place to test the efficacy of sequential immunotherapy using autologous NK cells and Hsp70-peptide TKD/IL-2 in patients with NSCLC (stage IIIa, b) after radiotherapy and chemotherapy (Table 1) [164]. It has been shown in a preclinical model of lung cancer that autologous NK cells-based therapies are highly effective against tumors selectively expressing membrane-type Hsp70 [193]. When combined with other ICIs or modulators, autologous NK cell therapy or other therapies that target tumors expressing mHsp70 may be effective in inhibiting tumor growth and progression [194]. In current NSCLC research, relay transfer of NK cells is being evaluated as a potential treatment method.

Furthermore, CAR-NK therapy has emerged as one of the most promising new research topics targeting NK cells for the treatment of NSCLC patients over the past few years [195]. CAR-NK cells have a number of benefits over CAR-T cells due to the fact that they can be produced from pre-existing cell lines or allogeneic NK cells with mismatched MHC. Moreover, they can kill cancer cells through CAR-dependent and CAR-independent pathways with less toxicity [196]. However, CAR-NK cells still have some limitations, such as the short half-life of NK cells and the extra-tumor toxicity [196,197]. Optimizing the CAR-NK structure and prolonging the survival of CAR-NK cells in vivo are the main research goals for the future.

The efficacy of NK cells immunotherapies in combination with other therapies has demonstrated promising results for NSCLC patients. The benefits of recently developed CAR-NK immunotherapies have also been recognized. However, immunotherapies associated with NK cells are still immature, and further research is needed to increase the treatment options for NSCLC patients.

## 4. Conclusions and Future Directions

The TME is engaged in the whole process of tumor growth and serves a crucial role. Different immune cells in the TME are required to mediate pro- and antitumor immune responses, which affect tumor destiny and the capacity for tumors to become aggressive and grow [21,198]. Due to the significance of immune cells in tumor progression, several immunotherapies have been designed for use in different types of cancer. Over the decades, there have been breakthroughs in immunotherapy, especially ICI immunotherapy applied to NSCLC patients, suggesting immunotherapy’s enormous potential for cancer treatment.

Anti-PD-1/PD-L1 immunotherapies are some of the most common ICIs. The FDA has authorized the representative PD-1- or PD-L1-blocking antibodies nivolumab, pembrolizumab, and atezolizumab for the treatment of NSCLC patients, and pembrolizumab has been used as a first-line treatment for patients with PD-L1 levels ≥ 1%. Recent clinical trial studies targeting PD-L1 include avelumab and durvalumab, and the most recent follow-up results for avelumab and the positive results shown with durvalumab in NSCLC patients after radiotherapy provide a potential treatment option [116]. In addition, it has been demonstrated that the anti-PD-1 drug cemiplimab substantially improved clinical outcomes in advanced NSCLC patients with more than 50% PD-L1 expression, indicating that this drug may be beneficial for NSCLC patients [116]. ICIs also contain antibodies against CTLA-4, such as ipilimumab. In the treatment of NSCLC, two-drug combinations of ICIs (e.g., nivolumab and ipilimumab) have the potential to be first-line therapies. Combination regimens of multiple ICIs offer additional options for future NSCLC patients. In addition, CAR-T genetically engineered T cell-related immunotherapy is being studied to improve the adverse effects seen in patients during the current trials as well as to make the treatment more effective for NSCLC patients in the future. For patients with EGRF-mutant NSCLC for whom ICIs therapy is ineffective, some EGRF-TKI drugs have been developed which have improved the survival rate among these patients. However, the long-term use of these drugs may result in a resistance; future research may focus on developing new drugs to reduce such resistance. TAMs-related immunotherapies are often used in combination with other immunotherapies to treat patients with NSCLC. ICIs with tazemetostat (an EZH2 inhibitor which targets TAMs) is an option for patients with NSCLC whose disease has progressed following first- or second-line therapy. Other TAMs-targeting cancer immunotherapies, such as MRX-2843, OR2805, and related nanotherapies, are currently undergoing clinical testing as promising NSCLC treatments. 

NSCLC patients with DC-related immunotherapies are mainly vaccinated with autologous DCs, and studies have shown that they are both safe and effective, though their exact efficacy remains unclear. 

Targeted NK cells therapies are mostly used in combination with other therapies for patients with NSCLC. The efficacy of NKTm cell transplantation, as well as cryosurgery combined with allogeneic NK immunotherapy, has been demonstrated. Autologous NK cells treatment or other medicines targeting mHsp70-expressing tumors, in conjunction with ICIs, may be beneficial in preventing the progression of NSCLC. Genetically engineered CAR-NK immunotherapies are being developed to improve NSCLC treatment in the future.

However, immunotherapy still has some drawbacks. ICIs boost T-cell activation in NSCLC patients by blocking negative regulators of T-cell function, which leads to an uncontrolled immune response and results in immune-related adverse events, including colitis, hypophysis, and pneumonitis [199]. Furthermore, checkpoint inhibitor pneumonia seems to be an uncommon but harmful side effect of anti-PD-1 and PD-L1 ICIs [193,200]. Thus, more scientific research and clinical studies are needed to improve treatment plans, reduce side effects, and control the immune response.

Moreover, sitravatinib (a TAM-targeting MerTK inhibitor) has exhibited an antitumor effect and modification of the TME in preclinical trials [201]. A retrospective analysis of NSCLC patients with tumor progression after ICI treatment showed that sitravatinib does not have significant antitumor activity when used singly. On the basis of its immunomodulatory effect on the TME, studies of sitravatinib in combination with ICI will be one of the primary areas of future research.

In summary, immune cells within the TME not only affect tumor growth and metastasis but are also used for therapy against tumors. For patients with advanced NSCLC, a number of immunotherapeutic strategies have been suggested as first-line therapies, and preclinical and clinical studies have proven their effectiveness. The combination of multiple medicines and the development of new therapeutic targets for NSCLC should be investigated in greater depth in the future, along with existing immunotherapy strategies, in order to increase the survival rate of cancer patients.

## Data Availability

Not applicable.
